# Germline de novo mutation rates on exons versus introns in humans

**DOI:** 10.1038/s41467-020-17162-z

**Published:** 2020-07-03

**Authors:** Miguel Rodriguez-Galindo, Sònia Casillas, Donate Weghorn, Antonio Barbadilla

**Affiliations:** 1 Centre for Genomic Regulation (CRG), The Barcelona Institute of Science and Technology, Dr. Aiguader 88, 08003 Barcelona, Spain; 2 Institute of Biotechnology and Biomedicine, Universitat Autònoma de Barcelona, Bellaterra (Cerdanyola del Vallès), 08193 Barcelona, Spain; 3 Department of Genetics and Microbiology, Universitat Autònoma de Barcelona, Bellaterra (Cerdanyola del Vallès), 08193 Barcelona, Spain; 4 Universitat Pompeu Fabra (UPF), Barcelona, Spain

**Keywords:** Computational models, Molecular evolution, Genetic variation, Mutagenesis

## Abstract

A main assumption of molecular population genetics is that genomic mutation rate does not depend on sequence function. Challenging this assumption, a recent study has found a reduction in the mutation rate in exons compared to introns in somatic cells, ascribed to an enhanced exonic mismatch repair system activity. If this reduction happens also in the germline, it can compromise studies of population genomics, including the detection of selection when using introns as proxies for neutrality. Here we compile and analyze published germline de novo mutation data to test if the exonic mutation rate is also reduced in germ cells. After controlling for sampling bias in datasets with diseased probands and extended nucleotide context dependency, we find no reduction in the mutation rate in exons compared to introns in the germline. Therefore, there is no evidence that enhanced exonic mismatch repair activity determines the mutation rate in germline cells.

## Abstract (web summary)

Evidence that somatic mutation rates in introns exceed those in exons challenges the molecular evolution tenet that mutation rate and sequence function are independent. Here, authors analyze germline de novo mutations and reveal no evidence for mutation rate differences between exons and introns.

## Introduction

One of the most general and widely accepted predictions of the neutral theory of molecular evolution is that “the more sequence conservation, the more functional (selective) constraint on the sequence”^1^. This principle explains why different functional regions in the genome have different levels of polymorphism and divergence, such as the lower variation at nonsynonymous vs synonymous sites in protein-coding genes or in exonic vs intronic sequences^2^. This relationship between constraint and variation constitutes one of the most powerful approaches in the current search for functional regions in the genome and the detection of natural selection at the molecular level. An integral part of estimating constraint, or purifying selection, on functional genomic regions is the comparison of the observed number of mutations to the expectation under neutral evolution. In genes, this neutral expectation is usually estimated from putatively nonfunctional regions or sites, including intronic sequence^3,4^. A main requirement for the validation of this assumption is that mutation rate on exons and introns does not correlate with that sequence function.

Mutation rate can vary strongly across the human genome, with regional differences up to threefold in the germline^5^ and at least up to fivefold in tumor cells^6^. It is influenced by several factors, including replication time, chromatin state, and expression level^6–8^. A priori, none of these factors are expected to correlate directly with genic sequence function (exonic vs intronic). Another important determinant of mutation rate is DNA sequence composition. Recent studies have addressed mutational processes and their associated sequence-dependent signatures, both in the soma and the germline^9–14^. Germline and many cancer tumor signatures exhibit a higher relative rate of C > T transitions for single nucleotide variants (SNV)^6,9,15^. Consequently, due to their higher G/C content, exonic regions show a context-driven relative increase in mutation rate compared to intronic regions^16^, which can be corrected for with the proper mutational model.

A differential mutation rate between intronic and exonic DNA beyond the context dependence would require a mutational process that recognizes the difference between the two functional sequence categories. Surprisingly, Frigola et al.^17^ found in tumoral DNA, primarily from skin melanomas and DNA-polymerase-*ε* (POLE)-mutant colorectal cancers, that mutation rates are lower in exons than in introns after accounting for the trinucleotide-context-dependent mutational signature. This reduced mutation rate in exons is similar both in synonymous and nonsynonymous sites, which rules out purifying selection as an explanation. The study suggests that the lower mutation rate in exons results from an enhanced mismatch repair (MMR) activity in exons compared to introns. In turn, the increased repair activity is attributed to different amounts of H3K36me3 epigenetic marks on exons and introns^17^.

In the germline, whether originating from replication errors or mediated by DNA damage, the dominant mutational processes are expected to produce mismatches^18–22^. Hence, any MMR-related mechanism is expected to play an important role in germline DNA damage repair. If the enhanced somatic exonic MMR activity found by Frigola et al.^17^ could be extrapolated to the germline, as the study suggests, then population and functional genomics studies would be compromised, and they should include differential exonic and intronic mutation rates as an integral part of their explanatory models.

Here, we investigate the relative mutation rates of exons and introns in the human germline using de novo mutation (DNM) data. We show that DNM densities do not differ between exons and introns after accounting for trinucleotide sequence composition and an excess of nonsynonymous exonic variation arising from sampling bias. We further explore factors that can impact DNM densities on exons and adjacent introns, namely extended sequence context dependency and several chromatin features, including H3K36me3 epigenetic marks. Finally, we provide estimates of exonic and intronic DNM rates.

## Results

### No evidence of reduced exonic DNM rate compared to introns

To study the distribution of germline mutations across exons and introns, we collected a total of 679,547 SNV DNMs from seven family-based WGS datasets, consolidating a high-density, high-quality DNM map across the human genome (see “Methods”). The compiled datasets show highly similar mutation spectra and an enrichment with CpG > TpG transitions (Supplementary Fig. 1). We first analyzed whether exonic and intronic mutation densities differ among human DNMs after accounting for sequence composition. For that purpose, we computed the observed total mutation burden at exonic and intronic sites by summing over 95,633 internal exon-centered sequences of size 2001 base pairs (bp), carrying a subset of 50,780 genic mutations. Since per-nucleotide mutation probability is influenced by the neighboring sequence context, we derived the expected mutation burden at each position of each 2001-bp internal exon-centered window from a context-dependent model (see “Methods”). We initially used a trinucleotide-context-dependent germline whole-genome mutation signature model, in line with the analysis presented in Frigola et al.^17^ for somatic mutations.

**Fig. 1:**
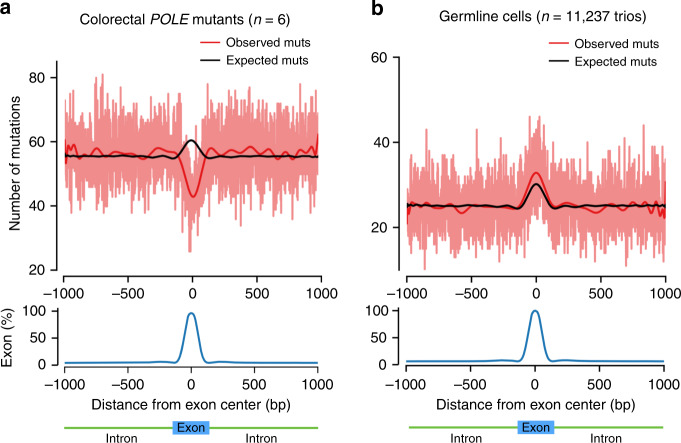
Internal exon-centered analyses on somatic and germline de novo mutations. Exon-centered 2001-nt-wide observed and expected mutational profiles (top) and exon density (bottom) in **a** somatic and **b** germline cells. The light red line represents the observed number of mutations at each position, whereas the dark red and black lines represent smoothed numbers of observed and expected mutations, respectively, obtained from a polynomial fit. **a** Profile of mutations in six *POLE*-mutant colorectal tumors, reprinted by permission from Springer Nature Customer Service Centre GmbH: Springer Nature Genetics, Reduced mutation rate in exons due to differential mismatch repair, Frigola et al.^17^. **b** Profile of mutations in the germline of 11,237 trios.

Frigola et al.^17^ found that the mutation burden of *POLE*-mutant tumors in positions dominated by exonic DNA is lower than expected (Fig. 1a). In contrast, in our study the observed germline exonic mutation burden was significantly increased by 7.2% (s.d. 1.4%; *P* = 0.001, permutation-based test) compared to the expectation across introns and exons (Fig. 1b). This result is robust to biases in mutation calling due to region mappability differences (Supplementary Table 1, Supplementary Fig. 2) and effects of transcription-coupled repair on the mutational pattern (Supplementary Fig. 3). This suggests that the hypothesized mechanism of enhanced repair on exons compared to introns in *POLE*-aberrant tumors is not determining mutation rate in the germline.

### Sampling bias explains exonic mutation density excess

Even in the absence of the supposed enhanced MMR effect on exonic DNA, we would expect a very slight deficit of exonic relative to intronic DNMs, due to strong purifying selection on lethal de novo variants in the early stages of embryonic development^23^. Therefore, we next investigated potential factors that could explain the observed increase in exonic relative to intronic mutation burden in DNM datasets, such as technical differences in sequencing and calling (Supplementary Table 2) and enrichment with diseased probands. As detailed in Table 1, the analyzed DNM datasets are heterogeneous regarding their study conditions, including disease cohorts. Diseased probands, e.g. those with autism spectrum disorder (ASD) or preterm birth, are more likely than average to carry mutations with functional impact^24,25^. This ascertainment bias in the data is expected to entail an enrichment with exonic nonsynonymous variants^26,27^. To test this, we classified the 4669 mutations in the internal exons as synonymous and nonsynonymous changes, resulting in 3488 nonsynonymous and 1170 synonymous DNMs (corresponding to a ratio of 2.98:1). We then repeated the internal exon-centered analysis for each of the two exonic mutation categories.

**Table 1:** Properties of analyzed DNM datasets including excess in exonic burden.

Dataset	Phenotypic condition	Control/proband DNMs	Exonic excess [%]	Emp. *p* value
Halldorsson^36^	Mixed (multiple diseases)	180,151	11.7 ± 2.8	0.001
Yuen^38^	Mostly ASD	127/117,612	8.0 ± 4.1	0.015
Goldmann^35^	Mixed (preterm birth)	35,793	13.0 ± 6.6	0.016
Sasani^39^	Random (mostly healthy)	27,454/0	12.3 ± 7.9	0.052
An^37^	ASD + healthy sibling	115,697/117,942	3.5 ± 2.2	0.070
GoNL^16^	Random (mostly healthy)	11,016/0	12.1 ± 14.5	0.181
Goldmann^29^	Healthy	73,755/0	2.5 ± 4.4	0.291
Autism probands	ASD	0/235,554	8.4 ± 2.5	0.001
Representative sample	Non-ASD + ASD	98,300/1,700	−2.5 ± 3.5	0.233
Healthy probands	Healthy	189,579/0	−0.5 ± 2.5	0.417

The seven used datasets and their references are shown above the line, while below the results for the composed datasets (see “Methods”) are given. In each group, datasets are ordered from most to least significant exonic mutation excess. Errors of the exonic excess denote 1 s.d. from 1000 permutations (see “Methods”).

**Fig. 2:**
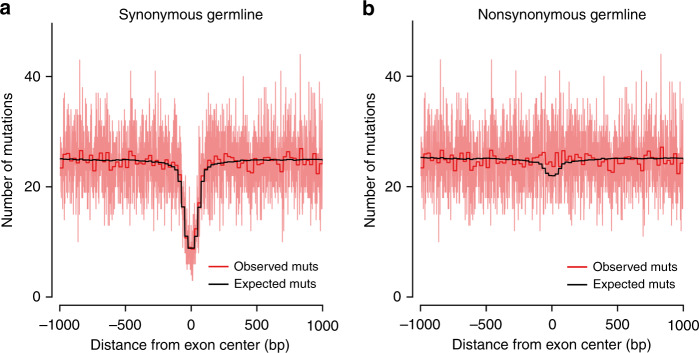
Internal exon-centered analysis for synonymous and nonsynonymous DNMs. The light red line represents the observed number of mutations at each nucleotide position, while the dark red and black lines represent averages in bins of size 25 positions for observed and expected mutations, respectively. **a** Synonymous DNM profile with observed exonic mutation difference of  −1.1% (*P* = 0.353). **b** Nonsynonymous DNM profile, showing an exonic excess of 10.4% (*P* = 0.001). Due to the removal of nonsynonymous and synonymous mutations in panels (**a**) and (**b**), respectively, the total number of exonic mutations relative to flanking introns is reduced. The number of mutations, when moving away from the center, converges to that of Fig. 1b.

Figure 2a shows that the observed synonymous profile matches the expected profile almost perfectly, with a slight nonsignificant deficit (−1.1%, *P* = 0.353). However, exonic nonsynonymous mutations show a large and statistically significant excess compared to the expectation under the trinucleotide-context model (10.4%, *P* = 0.001, Fig. 2b). Note that this stratification by functional mutation category entails a reduction of the number of both synonymous and nonsynonymous mutations relative to flanking introns across the window of stacked sequences. This is why the number of mutations, when moving outwards from the center, converges to that of Fig. 1b. Overall, Fig. 2 suggests that disease ascertainment during data acquisition may be responsible for the overall excess of 7.2% of exonic variants. Therefore, we next repeated the analysis for all seven DNM datasets individually, as well as for assembled samples with only healthy, only ASD, or a representative mixture of probands (see “Methods”). We found a significant exonic mutation excess only in cohorts with a high fraction of diseased probands or those assembled purely from diseased samples, while all cohorts with mostly or exclusively healthy probands show no signal (Table 1). Moreover, when we stratify exonic mutations by functional impact, we find a statistically significant excess only in nonsynonymous mutations among ASD individuals (Supplementary Table 3).

### De novo variants show extended-context dependency

**Fig. 3:**
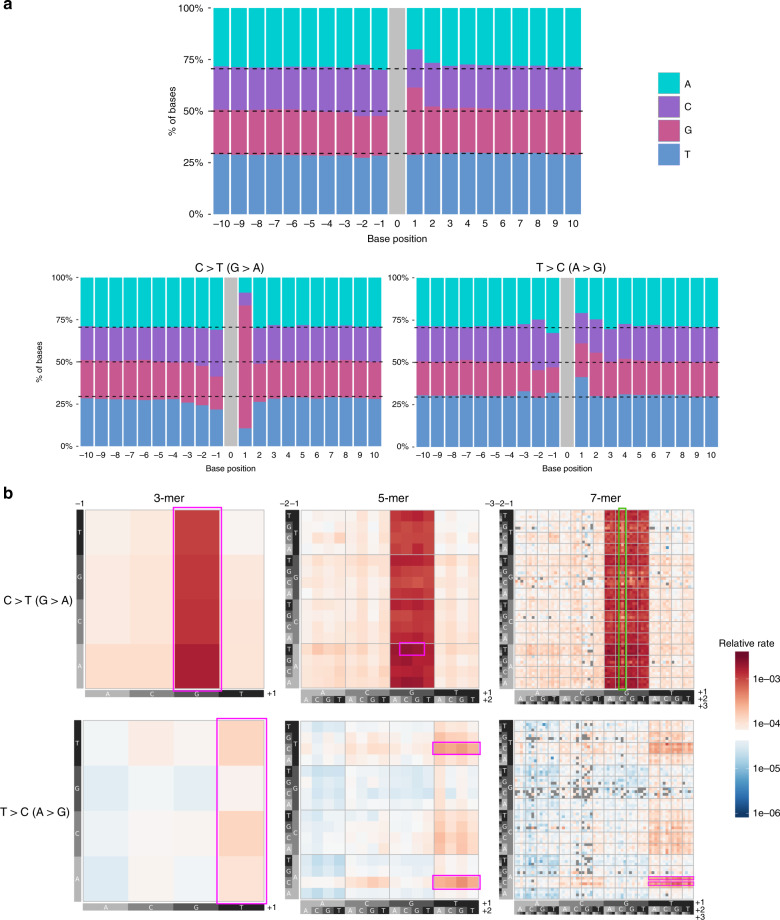
Mutational classes with highest sequence context dependency around de novo mutations. **a** Frequencies of nucleotides neighboring our entire set of 679,547 DNMs and subsets belonging to C > T (G > A) and T > C (A > G) 1-mer classes in a window of size 21 bp. Black dashed lines represent the whole-genome background frequencies for the four nucleotides. The extended sequence context dependency varies across 1-mer mutation classes. **b** Heatmap of estimated relative mutation rates, corrected by abundance of reference k-mer, for C > T (G > A) (top) and T > C (A > G) (bottom) 1-mer classes, up to a 7-mer resolution. For each 1-mer class, each of the three grids delineates mutation contexts of different length, defined by the upstream sequence (*y*-axis) and downstream sequence (*x*-axis) from the central (mutated) nucleotide. Boxed regions indicate motifs previously identified as hypermutable (pink) or hypomutable (green). Supplementary Figs. 4 and 5 show the corresponding plots for the other 1-mer classes.

The stratification into synonymous and nonsynonymous changes entailed a polarization of the exonic excess (Fig. 2), intensifying the signal for nonsynonymous variants (10.4%) with a concomitant decrease for synonymous variants (−1.1%). This type of polarization could be due to the incompleteness of our mutational model. Our mutational model for the expected number of mutations was constructed using trinucleotide-context-dependent mutation probabilities. However, it has been shown that SNPs segregating in the human population are affected by the extended flanking sequence, with a heptameric context explaining a majority of the observed mutation rate variability^13,14^. Figure 3 and Supplementary Figs. 4, 5 show that this is confirmed by DNMs based on the relative frequencies of all four nucleotides around mutations in our DNM dataset, although the effect of the extended flanking sequence is small compared to the one observed in *POLE*-mutated tumor genomes^28^.

We assessed the impact of context dependency by expanding our mutational signature model to incorporate the pentameric and heptameric mutational sequence context (based on exact computation and a likelihood decomposition approach, respectively; see “Methods”, Supplementary Fig. 6). We applied these extended-context models to the largest DNM dataset that had no diseased probands, Goldmann et al.^29^, as well as to this dataset and the pooled dataset stratified by synonymous and nonsynonymous variants. We found that while the overall likelihood increases for increasing context size, penalization due to the additional parameters of the larger context models entails that the trinucleotide-context-dependent model is found to be the best model for the current datasets based on the Akaike information criterion (AIC) (Table 2, Supplementary Tables 4–6).

**Table 2:** Extended sequence context dependency for Goldmann et al.^29^.

Model	Exonic excess [%]	Emp. *p* value	Log-likelihood	# param	AIC
1-mer	16.2 ± 5.4	0.001	−68,111	12	136,247
CpG	1.7 ± 4.3	0.348	−66,594	18	133,224
3-mer	2.7 ± 4.3	0.281	−66,315	192	133,014
5-mer	2.9 ± 4.6	0.258	−66,054	1344	134,797
7-mer	2.8 ± 4.3	0.266	−65,981	2496	136,955

Errors of the exonic excess denote 1 s.d. from 1000 permutations (see “Methods”).

### H3K36me3 does not correlate with exonic mutation density

**Fig. 4:**
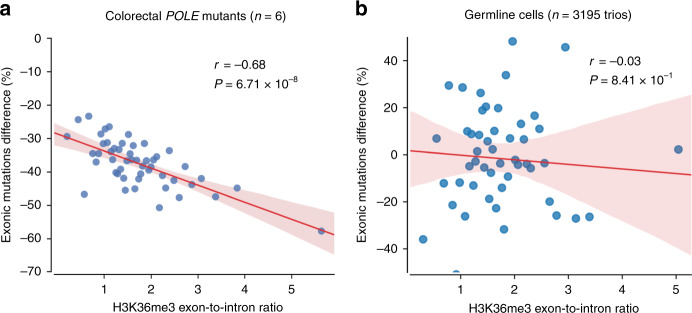
Deviation in the exonic mutation burden as a function of the H3K36me3 exon-to-intron ratio. Blue dots denote 50 groups of genes binned by their exon-to-intron ratio of H3K36me3 coverage (*x*-axis). The relative difference between the total observed and expected number of exonic mutations (computed using a 3-mer model) per group is shown on the *y*-axis. The trend line and its confidence interval were added using the seaborn package of Python, while the correlation coefficient and its significance were computed using the same iteratively re-weighted least-squares approach as used by Frigola et al.^17^ to ensure comparability. **a** *POLE*-mutant colorectal tumors. The H3K36me3 histone mark is derived from colonic mucosa (E075), reprinted by permission from Springer Nature Customer Service Centre GmbH: Springer Nature Genetics, Reduced mutation rate in exons due to differential mismatch repair, Frigola et al.^17^. **b** DNMs from healthy probands (a total of 3195 trios). The H3K36me3 histone mark is derived from H1 stem cells (E003).

The enhanced exonic MMR activity compared to introns in *POLE*-aberrant tumors was proposed to be mediated by the H3K36me3 mark^17^. Using our dataset of mutations from healthy probands, we therefore investigated the relative exonic mutation density as a function of H3K36me3 and nucleosome density. These two features show differential coverage between (mainly internal) exons and introns (Supplementary Fig. 7), and had been previously described to contribute to the recognition of splice marks at internal exon–intron boundaries^30,31^. We observed no significant correlation (*r* = −0.03, *P* = 0.84) between the exonic mutation enrichment and the H3K36me3 mark (Fig. 4b). This contrasts with the recruitment mechanism described in somatic cells^32^, which is invoked as the mechanistic hypothesis behind the findings in Frigola et al.^17^ (Fig. 4a). Conversely, we find a negative, nearly significant correlation (*r* = −0.28, *P* = 5.38 × 10^−2^) with nucleosome coverage (Supplementary Fig. 8). This result complements the previously reported influence of nucleosome organization on human germline DNMs^5,33^.

### Estimation of exonic and intronic de novo mutation rate

We estimated germline DNM rates for exons and introns separately. Using the largest dataset with only healthy probands^29^, we estimated 1.38 × 10^−8^ and 1.11 × 10^−8^ mutations per site per generation for exons and introns, respectively. This difference reflects the higher mutability of exons with respect to introns due to sequence differences, namely higher CpG and overall GC content^16^. These estimates are consistent with a previously reported whole-genome based rate of 1.2 × 10^−8^ mutations per site per generation^20^. Conversely, estimates obtained from the pooled dataset reflect the disease ascertainment bias, with a similar intronic mutation rate (1.15 × 10^−8^), but a much larger exonic rate (1.52 × 10^−8^), in line with previous findings in diseased cohorts^34^.

## Discussion

We compiled human DNM data to show that the rate of generation of new genetic variants, the mutation rate, does not significantly vary between exons and adjacent introns when accounting for sequence context. Moreover, we went beyond previous analyses that used extreme rare variants as a proxy for DNMs^13,14^ and described directly germline mutation patterns based on a large aggregated DNM dataset. We corroborated earlier findings, in particular an extended-context dependence of germline variants. At the same time, the internal exon-centered analysis, with its relatively low number of mutations compared to the entire dataset, is still adequately described by a trinucleotide-context-dependent model. Beyond context dependence, the sampling bias introduced by enrichment with diseased probands is one of the most important confounding factors of DNM analyses. We showed that its effects can lead to significant deviations from the null model and, depending on the application, should be addressed through an informed choice of samples.

Our analysis shows that the results found in the soma cannot be directly extrapolated to the germline, and the MMR-dependent process that was proposed as an explanation for the decreased exonic mutation burden in somatic cells does not seem to determine germinal cell mutation rates. Last, this study provides a clear-cut answer to the challenge posed by Frigola et al.^17^: It validates the main assumption of molecular population genetics—that genomic mutation rate does not depend on sequence function—and demonstrates different mutational dynamics in somatic vs germinal cells.

## Methods

### De novo mutation data

We aggregated DNMs from seven family-based WGS datasets coming from multiple centers and projects: the Genomes of the Netherlands (GoNL) project^16^, the Inova Translational Medicine Institute Preterm Birth Study^35^, Inova Translational Medicine Institute’s Longitudinal Childhood Genome Study^29^, deCODE genetics^36^, Simons Simplex Collection (SCC) and Korean ASD cohort^37^, the Autism Genetic Research Exchange (AGRE) repository^38^ and Centre d’Etude du Polymorphisme Humain (CEPH)^39^. Mutation datasets were downloaded from the supplementary tables of the respective papers^35–39^ or by direct request to the authors^29^. Data from the GoNL were downloaded from http://www.nlgenome.nl. Most of datasets were originally mapped to hg19, with exception of the data from An et al.^37^ and Halldorson et al.^36^, which were mapped to hg38. Subsequently, coordinates in these datasets were lifted over to hg19, the most common reference genome in our data. To avoid possible biases arising from mutation calling on sexual chromosomes, only autosomal SNVs were used, leaving a total of 679,547 germline SNV DNMs coming from 11,237 trios.

### Effect prediction of de novo mutations

The predicted consequence class of all DNMs was obtained using the Ensembl Variant Effect Predictor (VEP)^40^ for the GRCh37/hg19 assembly. Since some DNMs were reported to have more than one consequence, e.g. different transcripts or overlapping genes, only one predicted consequence for each DNM was retrieved (according to VEP criteria). Predictions are classified from major to mild according to the Ensembl Variation hierarchy.

### Whole-genome de novo mutation spectrum

All mutations were divided into nine classes, considering the fact that CpG sites are highly mutagenic. The number of mutations were corrected by the relative abundance of the context in the whole genome, e.g. the total number of C > T (G > A) transitions occurring at CpG sites divided by the relative abundance of CpG sites in the genome. We performed the mutational analysis across all used studies (Supplementary Fig. 1). For all subsequent analyses, extended nucleotide-context-dependent mutational models were used.

### Genomic coordinates of internal exons and flanking introns

Coordinates for a total of 20,345 protein-coding genes were obtained from GENCODE v19 ^41^. Genes without introns and overlapping genes were discarded, leaving a filtered set of 13,474 genes. Genes located on chromosomes X, Y and on the mitochondrial genome were removed from the analysis, leaving a total of 12,754 autosomal genes. Finally, all transcripts per gene were merged into meta-exon and meta-intron coordinates, both 5′ and 3′ flanking exons were removed as well as UTRs. Only internal exons (unfiltered by mappability issues, see below) were used for the main internal exon-centered mutational analyses.

Moreover, positions where mutation calling would be technically challenging because of mappability issues were removed, leaving a total of 10,237 genes for the gene by gene analyses. We also filtered out the internal exon-centered 2001-nt windows that overlapped at least one nucleotide with regions with mappability issues (Supplementary Table 1) for the supplementary internal exon-centered analysis restricted to highly mappable regions (Supplementary Fig. 2). Coordinates of unreliable regions^42^ were obtained from the UCSC Genome Browser, available at http://genome.ucsc.edu/cgi-bin/hgFileUi?db=hg19&g=wgEncodeMapability.

Meta-exon and meta-intron coordinates of genes with at least five meta-exons (not only internal) were extracted from GENCODE v19 for the analyses with chromatin features across genic regions (Supplementary Fig. 7).

### Sequence context model

We directly computed the probability of a mutation into an alternative nucleotide, *H*_*a*_ where *a* ∈ {1, 2, 3}, given the reference nucleotide *H*_r_ and its flanking sequence ${\bf{X}}=({{\bf{X}}}_{5^{\prime} },{{\bf{X}}}_{3^{\prime} })$, where ${{\bf{X}}}_{5^{\prime} }$ and ${{\bf{X}}}_{3^{\prime} }$ are the 5′ and 3′ flanking sequences, respectively. Here, *H*_r_ ∈ **M** = {A, C, G, T}, where the latter denote nucleotides adenine, cytosine, guanine, and thymine, and ${H}_{a}\in {\bf{M}}^{\prime} =\{m\in {\bf{M}}\,,m\,\ne\, {H}_{\text{r}}\}$. For example, for the 5-mer GCACG > GCTCG mutation *H*_r_ = A, *H*_*a*_ = T, ${{\bf{X}}}_{5^{\prime} }=(\,\text{G},\text{C}\,)$, ${{\bf{X}}}_{3^{\prime} }=(\,\text{C},\text{G}\,)$ and **X** = (G, C, C, G). Therefore, the probability of each of the possible k-mer changes, normalized by the abundance of each reference k-mer in the genome, was computed as follows:

1 $$P({H}_{a}| {H}_{\mathrm{r}},{\bf{X}})=\frac{N({H}_{a},{H}_{\text{r}},{\bf{X}})}{G({H}_{\text{r}},{\bf{X}})},$$

where *N*(*H*_*a*_, *H*_r_, **X**) is the genome-wide number of observed mutations into alternate allele *H*_*a*_ with given reference allele *H*_r_ and flanking sequence **X**. *G*(*H*_r_, **X**) is the abundance of the reference k-mer with reference nucleotide *H*_r_ and flanking sequence **X** in the genome. We computed the relative abundance of each reference k-mer in the autosomal genome using the pyFasta package. We also computed strand-wise signatures restricted to mutations falling in genic regions (exons at the canonical CDS and the respective introns) for the supplementary analysis in Supplementary Fig. 3. To compute strand-wise signatures, we polarized mutations according to the transcription strand on which the canonical CDS is annotated.

For some k-mer models, given limitations imposed by the amount of DNMs, we used a decomposition approach to compute the probability. For a k-mer model of sequence length *k*, let *H*_r_ be a reference core h-mer of length *h* and *H*_*a*_ an alternate core h-mer, where 1 ≤ *h* < *k*. Let the tuple **X** = (*x*_1_, …, *x*_*g*_) with *g* = (*k* − *h*) elements represent again the flanking sequence of the core h-mer, where *x*_*i*_ ∈ **M** ∀*i* ∈ {1,…, *g*}. In other words, **X** ∈ **M**^*g*^ where **M**^*g*^ is the *g*-fold Cartesian product. For example, with *k* = 7, *h* = 3 and the mutation ACTGACT > ACTCACT, then *H*_r_ = TGA, *H*_*a*_ = TCA and **X** = (*x*_1_ = A, *x*_2_ = C, *x*_3_ = C, *x*_4_ = T). We then approximate the probability *P*(*H*_*a*_∣*H*_r_, **X**) by:

2 $$P({H}_{a}| {H}_{\text{r}},{\bf{X}})\approx P({H}_{a}| {H}_{\text{r}})\cdot \mathop{\prod }\limits_{i = 1}^{g}\frac{{P}_{i}({H}_{a}| {H}_{\text{r}},{x}_{i})}{P({H}_{a}| {H}_{\text{r}})},$$

with

3 $$P({H}_{a}| {H}_{\text{r}})=\frac{{\sum }_{{\bf{Z}}\in {{\bf{M}}}^{g}}N({H}_{a},{H}_{\text{r}},{\bf{Z}})}{{\sum }_{{\bf{Z}}\in {{\bf{M}}}^{g}}G({H}_{\text{r}},{\bf{Z}})},$$

and

4 $${P}_{i}({H}_{a}| {H}_{\text{r}},{x}_{i})=\frac{{\sum }_{{\bf{Z}}\in {{\bf{Y}}}_{{x}_{i}}}N({H}_{a},{H}_{\text{r}},{\bf{Z}})}{{\sum }_{{\bf{Z}}\in {{\bf{Y}}}_{{x}_{i}}}G({H}_{\text{r}},{\bf{Z}})}\ ,$$

where

5 $${{\bf{Y}}}_{{x}_{i}}=\left\{({y}_{1},...,{y}_{j},...,{y}_{g})\ \ \ \ \forall j\in \{1,...,g\}\left\{\begin{array}{l}\,\text{if}\,\,j=i,\,{y}_{j}={x}_{i}\quad \\ \,\text{otherwise}\,,\,{y}_{j}\in {\bf{M}}\quad \end{array}\right.\right\}\ .$$

We implemented this framework using custom Python code. The composite likelihood model was applied to the 7-mer analysis of the data pooled across all cohorts using *k* = 7 and *h* = 5. Also in the analysis of the largest single dataset purely composed of healthy probands^29^ and the largest single dataset^36^, in each for 5-mers with *k* = 5 and *h* = 3 and for 7-mers with *k* = 7 and *h* = 3. The rest of probabilities were computed using the direct approach. Supplementary Fig. 6 shows the relationship between the exact computations of mutational probabilities and the composite likelihood model for the pooled dataset.

The number of parameters for the direct approach increases exponentially as *k* increases following *f*(*x*) = 4^*x*^ ⋅ 3, where *x* = *k*. For the decomposition approach they increase linearly as *k* − *h* increases from a fixed *h* following *g*(*y*, *x*) = *f*(*x*) ⋅ (1 + 3(*y* − *x*)), where *y* = *k* and *x* = *h*.

### Comparison of sequence context models

We selected the sequence context dependency model that best explains mutations across our set of exonic and intronic sequences by means of the AIC. We interrogated each of the 191,361,633 (~6.4% of the whole-genome length) exonic and intronic sites on the 95,633 2001-nt windows for the state in the observed data: mutated (and type) or not mutated. For recurrent sites, we chose one observed mutation at random. For a given model, we computed the log-likelihood as the sum across sites of the logarithm of the estimated probability of the observed state at the site. Probabilities were estimated with mutations from the entire dataset, through the direct or the decomposition approach as stated above.

### Internal exon-centered mutational analysis

A total of 95,633 stacked 2001-nt sequences centered on the middle position of internal meta-exons were used to compare the observed and expected mutational profiles across exons and introns. We computed the frequency of mutation at a site *l* with reference core sequence ${H}_{\,\text{r}\,}^{l}$ and flanking sequence **X**^*l*^ as

6 $${f}_{l}=\mathop{\sum }\limits_{a = 1}^{3}P({H}_{a}^{l}| {H}_{\,\text{r}\,}^{l},{{\bf{X}}}^{l}),\qquad l\in \{1,...,L\}\ ,$$

where *L* = 2001 denotes the total number of considered sites. Then, each frequency was normalized by the total frequency on the sequence:

7 $${f}_{l}^{\,\text{resc}\,}=\frac{{f}_{l}}{\mathop{\sum }\nolimits_{l^{\prime} = 1}^{L}{f}_{l^{\prime} }}\ .$$

Finally, the total number of observed mutations *n*_*s*_ on each of the 2001-nt sequence *s*, of a total of *S* = 95,633 stacked sequences, was redistributed across both middle exonic and flanking intronic sites according to the normalized frequencies:

8 $${\hat{n}}_{s}^{l}={f}_{l}^{\,\text{resc}\,}\cdot {n}_{s},\qquad s\in \{1,...,S\}\ ,$$

thus yielding the expected number of mutations at site *l* of a given sequence *s*. By adding up the values of all the stacked sequences, we obtain the cumulative number of expected mutations at site *l*,

9 $${\hat{n}}^{l}=\mathop{\sum }\limits_{s = 1}^{S}{\hat{n}}_{s}^{l}\ .$$

For the internal exon-centered analysis on synonymous or nonsynonymous mutations, we separated all possible exonic mutations in middle exon sequences into two groups: those with synonymous consequence and those with a consequence ranking higher than synonymous in the Ensembl Variation hierarchy. Then we computed the expected numbers by only adding frequencies for either synonymous or nonsynonymous mutations.

### Computation of effect size and statistical significance

We performed 1000 random permutations of the observed mutations in each stacked sequence based on the probability of each site to acquire a mutation. The effect size, defined as the relative increase or decrease in observed exonic mutations with respect to the expected number, was computed based on the simulation mean expected value. The error of this estimate is given as one standard deviation derived from the 1000 permutations. Moreover, we computed an empirical one-sided *p* value as the fraction of the simulations with more (or fewer) exonic mutations than the observed number of exonic mutations.

### Composed datasets

Mutations were only resampled from datasets with known conditions of the probands, either from healthy probands or those with ASD. Given an ASD prevalence in humans of 1.7%, we created a random sample of 100,000 whole-genome DNMs, taking 98,300 mutations classified as strictly from healthy probands and 1700 classified as ASD and repeated the internal exon-centered analysis. To generate the purely healthy and purely ASD cohorts, we used solely mutations from probands with the respective condition.

### Nucleosome and H3K36me3 histone mark data

We downloaded narrow peak coordinates and genome-wide read-coverage of H3K36me3 from human embryonic stem cell H1-hESC (E003), as proxy for germline cells, from the Epigenome Roadmap consortium^43^ data portal (http://www.roadmapepigenomics.org/data). The genome-wide nucleosome positioning density graph of ENCODE^44^ cell line GM12878 (lymphoblastoid cell line) was obtained via the UCSC genome browser (https://hgdownload.soe.ucsc.edu/downloads.html). Nucleosome peak regions were identified across the genome by using the bwtool program (with parameters local-extrema -maxima -min-sep = 150). The window of 146 bp flanking the peak coordinate (73 bp per side) was considered the region covered by a nucleosome.

### Coverage of chromatin features across exons and introns

Exons and introns in each gene were classified according to their position with respect to the transcription start site, where the ones that occupy different positions in different transcripts were discarded. We also discarded exons and introns at the lower quartile of length to compute the coverage for a set of exons or introns of heterogeneous lengths in a given position: the fraction of bases covered by H3K36me3 and nucleosomes at the center of the stack corresponding to the window defined by the shortest exon or intron remaining after the filtering. Finally, the difference between the exonic and intronic coverage was computed via the two-tailed Mann–Whitney *p* value of the comparison of both distributions.

We also computed the positions in the genome covered by H3K36me3 or nucleosomes across 95,633 internal exon-centered 4001-nt windows. By stacking sequences, we obtained middle exon-centered profiles of coverage across exons and introns (Supplementary Fig. 7).

### Nucleosome and H3K36me3 binned gene analysis

For each gene, we computed the readcount-based exonic enrichment of H3K36me3 or nucleosomes as the ratio between the exonic and intronic total number of bases covered by reads of the chromatin feature. Genes with no exonic and intronic bases covered by reads were removed from the analysis, as well as genes without any observed exonic or intronic mutation. Thus, a total of 7215 and 6529 genes remained for the H3K36me3 and nucleosome analysis, respectively.

For a given gene, we computed the exonic expected number of mutations as follows:

10 $${\hat{n}}_{\text{e}}={P}_{\text{e}}\cdot n\ ,$$

where *n* is the total number of mutations (both exonic and intronic) observed on the gene and *P*_e_ is the (binomial) probability of a mutation to fall on the exonic region of the gene, which in turn is computed as:

11 $${P}_{\text{e}}=\frac{{{\mathcal{L}}}_{\text{e}}}{{{\mathcal{L}}}_{\text{e}}+{{\mathcal{L}}}_{\text{i}}}\ ,$$

with

12 $${{\mathcal{L}}}_{\text{e}}=\mathop{\sum }\limits_{{l}_{\text{e}} = 1}^{{L}_{\text{e}}}\mathop{\sum }\limits_{a = 1}^{3}P({H}_{a}^{{l}_{\text{e}}}| {H}_{\text{r}}^{{l}_{\text{e}}},{{\bf{X}}}^{{l}_{\text{e}}})\ ,$$

and

13 $${{\mathcal{L}}}_{\text{i}}=\mathop{\sum }\limits_{{l}_{\text{i}} = 1}^{{L}_{\text{i}}}\mathop{\sum }\limits_{a = 1}^{3}P({H}_{a}^{{l}_{\text{i}}}| {H}_{\text{r}}^{{l}_{\text{i}}},{{\bf{X}}}^{{l}_{\text{i}}})\ .$$

Here, *l*_e_ ∈ {1,…, *L*_e_} and *l*_i_ ∈ {1,…, *L*_i_} denotes the set of all exonic and intronic positions of a given gene, respectively. ${{\mathcal{L}}}_{\text{e}}$ and ${{\mathcal{L}}}_{\text{i}}$ represent the exonic and intronic target size, respectively, expressed as the sum of the probability $P({H}_{a}^{l}| {H}_{\,\text{r}\,}^{l},{{\bf{X}}}^{l})$ of all possible three mutations that can happen across all exonic or intronic sites of the gene. The probability was computed under a 3-mer model for each of the genes as explained above only with mutations from the composed dataset of healthy probands.

Afterwards, genes were grouped into 50 bins according to their exonic enrichment of H3K36me3 or nucleosomes. Then, with the observed *n*_e_ and expected ${\hat{n}}_{\text{e}}$ exonic mutations over all genes in the bin, we computed the relative difference between the observed and expected number of exonic mutations per bin as follows:

14 $$\,{ {\mathrm{Exonic}}\,\, {\mathrm{mutations}} \,\,{\mathrm{difference}} \,[\%]}=\frac{{n}_{\text{e}}-{\hat{n}}_{\text{e}}}{{\hat{n}}_{\text{e}}}\cdot 100\ .$$

Finally, we computed the correlation between the median exonic chromatin feature enrichment and the difference in exonic mutations across the bins. The trend line and its confidence intervals were added using the bootstrapping functions of the python seaborn package, which confers equivalent weights in the regression to all points. In order to guarantee that the trend is not the result of a few outliers, the correlation coefficient and its significance were computed using an iteratively re-weighted least-squares approach, letting the variance of exonic chromatin feature enrichment of the bins influence the weight of each point.

### Estimation of absolute mutation rates

We estimated absolute mutation rate in our set of 95,633 middle exons as a proxy of mean exonic mutation rate and absolute mutation rate on the rest of the 2001-nt window as a proxy of mean intronic mutation rate as follows:

15 $$\mu =\frac{{N}_{\text{obs}}}{{N}_{\text{site}}\cdot {N}_{\text{gen}}}\ .$$

Here, *μ* is the mutation rate per site and generation, *N*_obs_ is the number of observed mutations, *N*_site_ is the number of sites (we used *N*_site_ = 13,632,264 exonic sites and *N*_site_ = 177,729,369 flanking intronic sites) and *N*_gen_ is the number of generations. A total of *N*_gen_ = 2582 gametogeneses for the largest dataset with healthy probands^29^ and *N*_gen_ = 22,474 gametogeneses in the pooled dataset.

### Reporting summary

Further information on research design is available in the Nature Research Reporting Summary linked to this article.

## Supplementary information

Supplementary Information

Peer Review

Reporting Summary

## Data Availability

All the analyses in this study were based on published datasets. Mutation data from the Genomes of the Netherlands (GoNL) project^16^ was downloaded from (http://www.nlgenome.nl). The remaining mutation datasets were either by direct request to the authors^29^ or downloaded from the supplementary tables of their respective publications^35–39^. Coordinates of unreliable regions^42^ were obtained from the UCSC Genome Browser, available at http://genome.ucsc.edu/cgi-bin/hgFileUi?db=hg19&g=wgEncodeMapability. Narrow peak coordinates and genome-wide read-coverage of H3K36me3 from human embryonic stem cell H1-hESC (E003) were downloaded through the Epigenome Roadmap consortium^43^ data portal (http://www.roadmapepigenomics.org/data). The genome-wide nucleosome positioning density graph of ENCODE^44^ cell line GM12878 (lymphoblastoid cell line) was obtained via the UCSC genome browser (https://hgdownload.soe.ucsc.edu/downloads.html).

## Code availability

Custom scripts and associated files needed to reproduce all analyses described here are provided together with the code at https://bitbucket.org/weghornlab/germline_intron_exon_mutrate. Segments of the code are based on scripts shared by Frigola et al.^17^ to ensure comparability of the main analysis.
