# Supplementary material for: Germline de novo mutation rates on exons versus introns in humans
Source: Nat Commun. 2020 Jul 3;11:3304. doi: 10.1038/s41467-020-17162-z (PMC7334200; doi:10.1038/s41467-020-17162-z)
Supplement: Supplementary file 1 — Supplementary Information [file 41467_2020_17162_MOESM1_ESM.pdf]

Germline *de novo* mutation rates on exons versus introns in humans

Rodriguez-Galindo et al.

— Supplementary Information —

Supplementary Figures

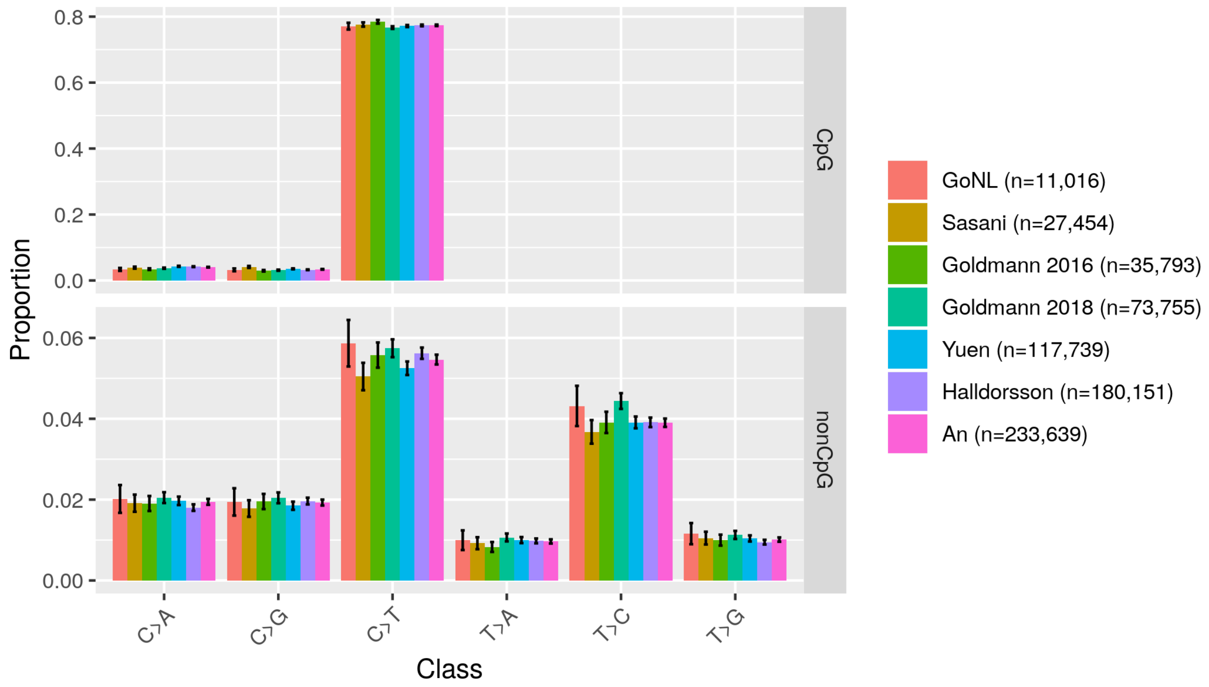

Supplementary Figure 1. Mutation spectrum across nine mutation classes for all analyzed DNM datasets. Datasets are by order GoNL[1], Sasani et al.[2], Goldmann et al. (2016)[3], Goldmann et al. (2018)[4], Yuen et al.[5], Halldorsson et al.[6] and An et al.[7]. Error bars denote binomial confidence intervals at level $\alpha = 0.01$, i.e. $2.33\sqrt{n_c/n(1-n_c/n)/n}$ for mutation class count n_c and total mutation count n.

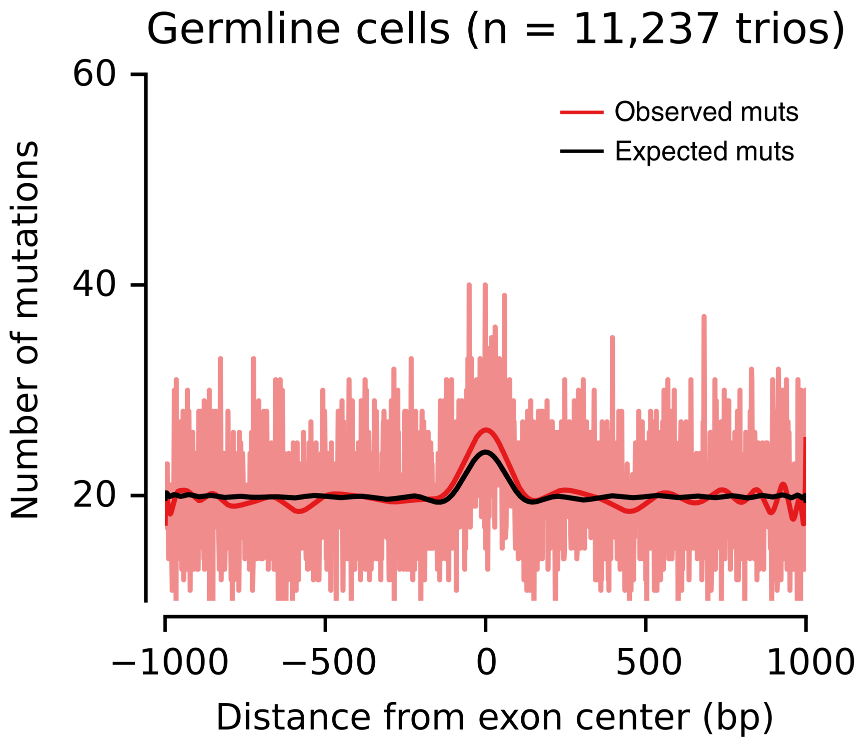

Supplementary Figure 2. Internal exon-centered analyses on germline *de novo* mutations restricting to highly mappable regions. Exon-centered 2,001-nt-wide observed and expected mutational profiles. The light red line represents the observed number of mutations at each position, whereas the dark red and black lines represent smoothed numbers of observed and expected mutations, respectively, obtained from a polynomial fit. The observed germline exonic mutation burden is significantly increased by 6.6% (s.d. 1.7%; P=0.001, permutation-based test) compared to the expectation across introns and exons.

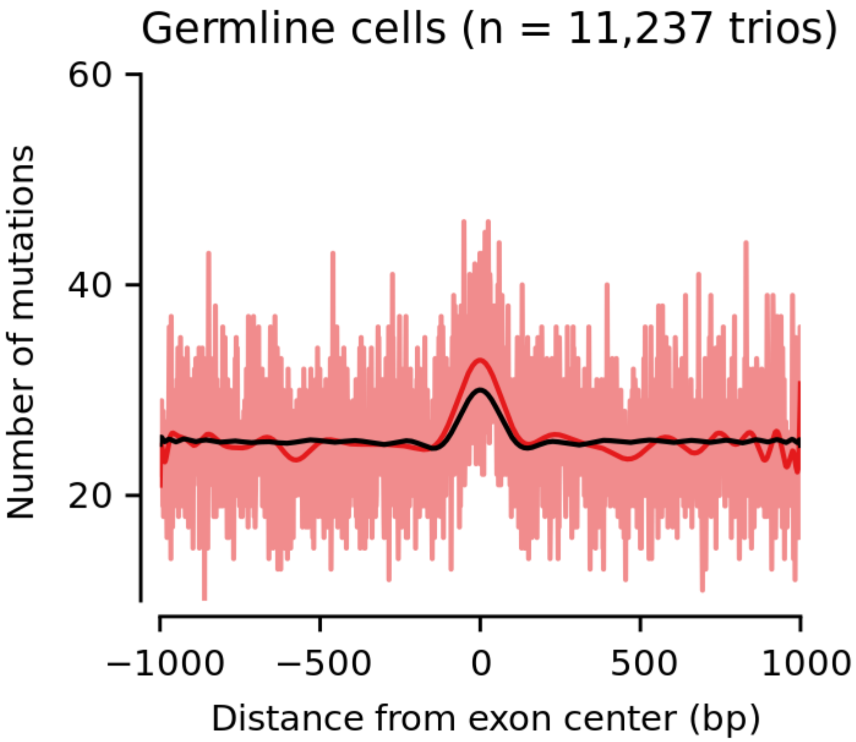

Supplementary Figure 3. Internal exon-centered analyses on germline *de novo* mutations restricting the mutational model to genic regions. Exon-centered 2,001-nt-wide observed and expected mutational profiles. The light red line represents the observed number of mutations at each position, whereas the dark red and black lines represent smoothed numbers of observed and expected mutations, respectively, obtained from a polynomial fit. Expected was computed based on a mutational model derived only from genic regions, to test the importance of transcription-coupled repair for the mutational signature. The observed germline exonic mutation burden is significantly increased by 7.8% (s.d. 1.5%; P=0.001, permutation-based test) compared to the expectation across introns and exons.

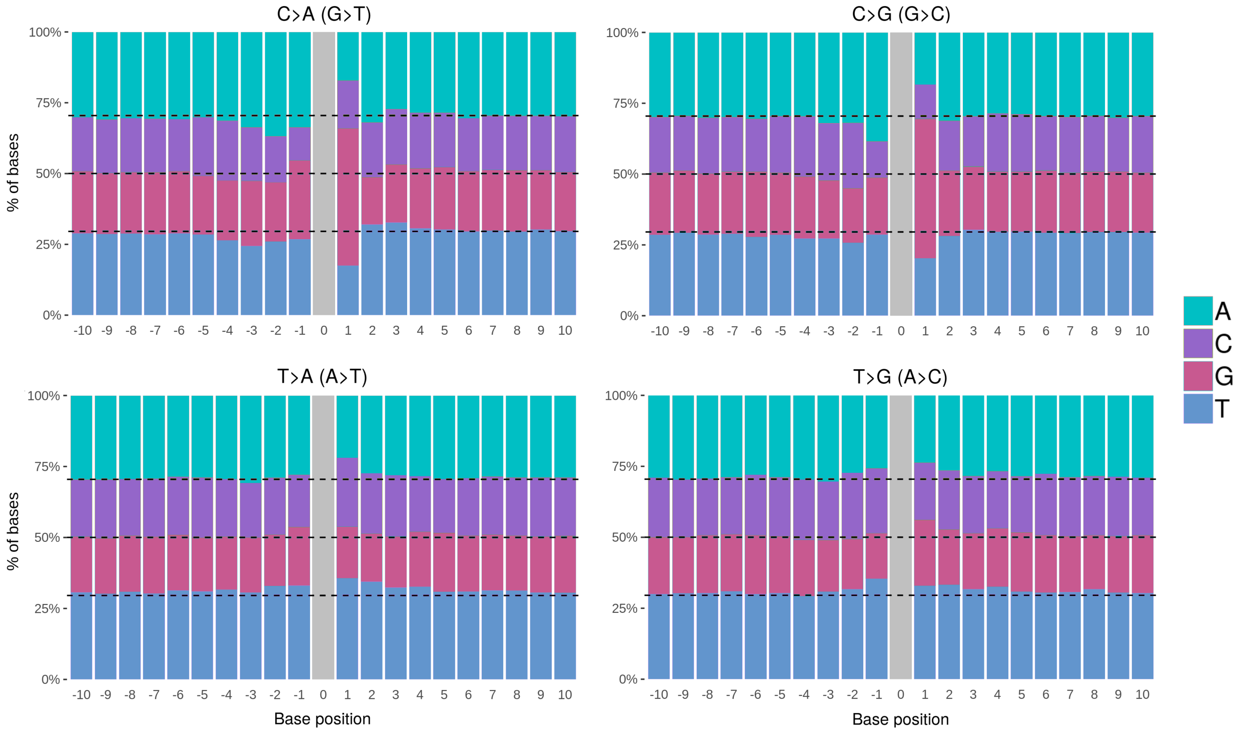

Supplementary Figure 4. Bar plot of sequence context around *de novo* mutations. Frequencies of nucleotides neighboring subsets of DNMs belonging to C>A (G>T), C>G (G>C), T>A (A>T) and T>G (A>C) 1-mer classes in a window of size 21 bp. Black dashed lines represent the whole genome background frequencies for the four nucleotides. The extended sequence context dependency varies across 1-mer mutation classes. This figure complements Figure 3a.

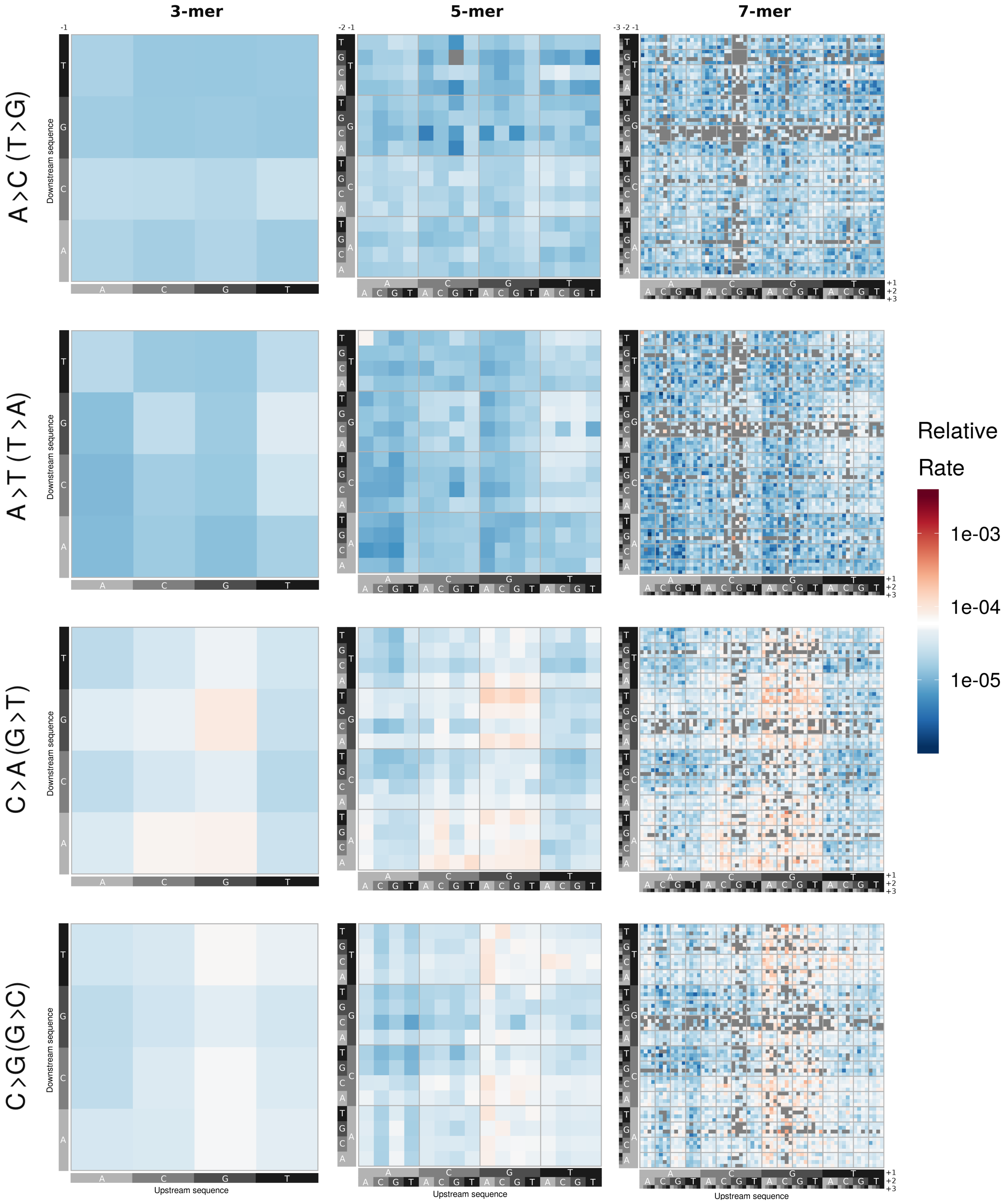
3-mer
5-mer
7-mer
A>C (T>G)
A>T (T>A)
C>A (G>T)
C>G (G>C)
Downstream sequence
Upstream sequence
Relative
Rate
1e-03
1e-04
1e-05

Supplementary Figure 5. Heatmap of sequence context around *de novo* mutations. Heatmap of estimated relative mutation rates for C>A (G>T), C>G (G>C), T>A (A>T) and T>G (A>C) 1-mer classes, up to a 7-mer resolution. For each 1-mer class, each of the three grids delineates mutation contexts of different length, defined by the upstream sequence (y-axis) and downstream sequence (x-axis) from the central (mutated) nucleotide. This figure complements Figure 3b.

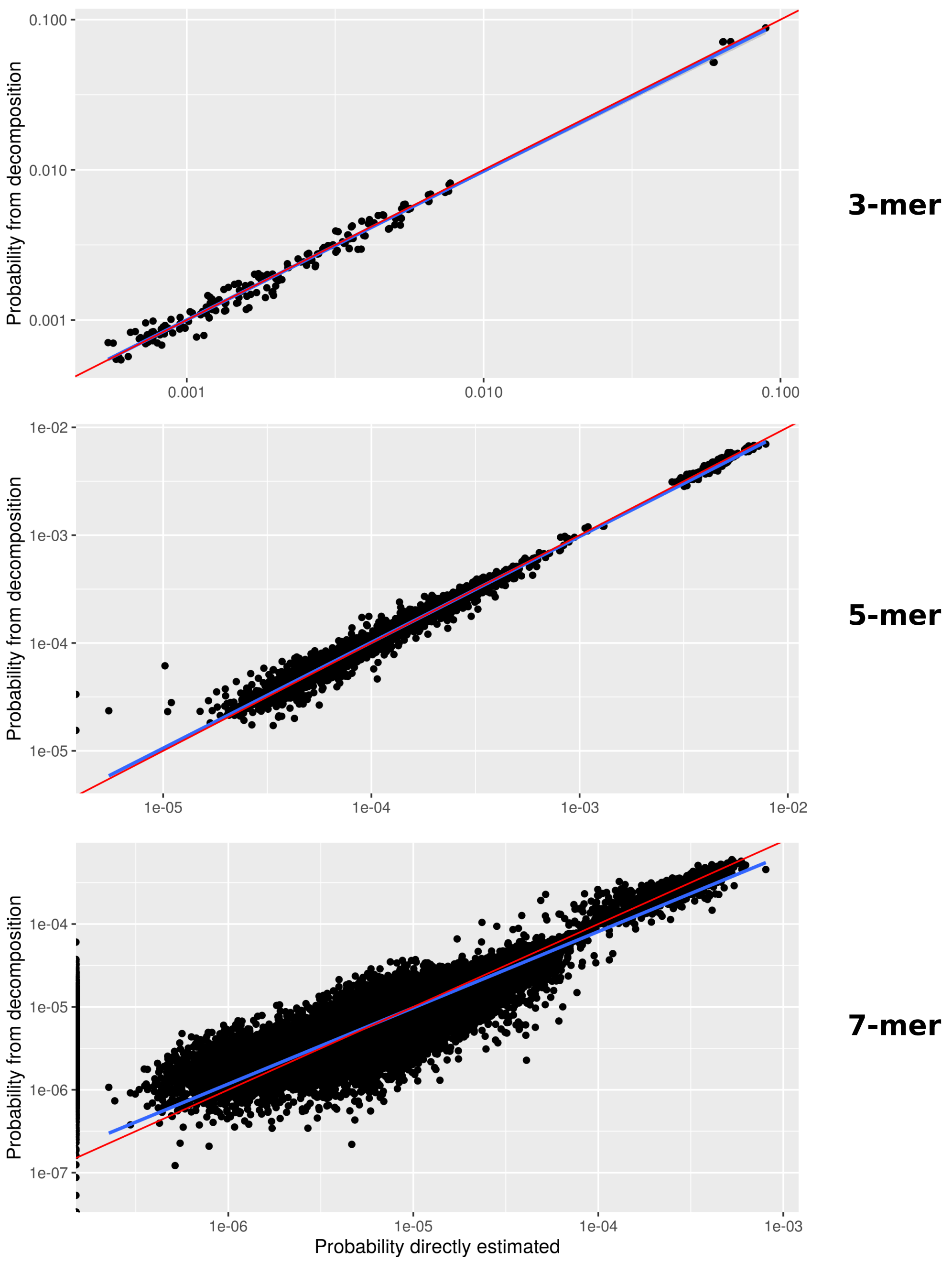

Supplementary Figure 6. Comparison between mutation probabilities obtained from a direct vs the composite likelihood approach. Each dot corresponds to a mutation. Pearson correlation coefficients are $\rho = 0.9966, \rho = 0.9979, \rho = 0.9858$ for 3-mer, 5-mer and 7-mer, respectively. Note that the linear regression (blue line) moves further away from the diagonal (red line) as the number of values that cannot be estimated through the direct method increases (e.g. for 7-mers 1,178 out of 49,152 mutational subtypes were erroneously estimated to have probability equal to zero).

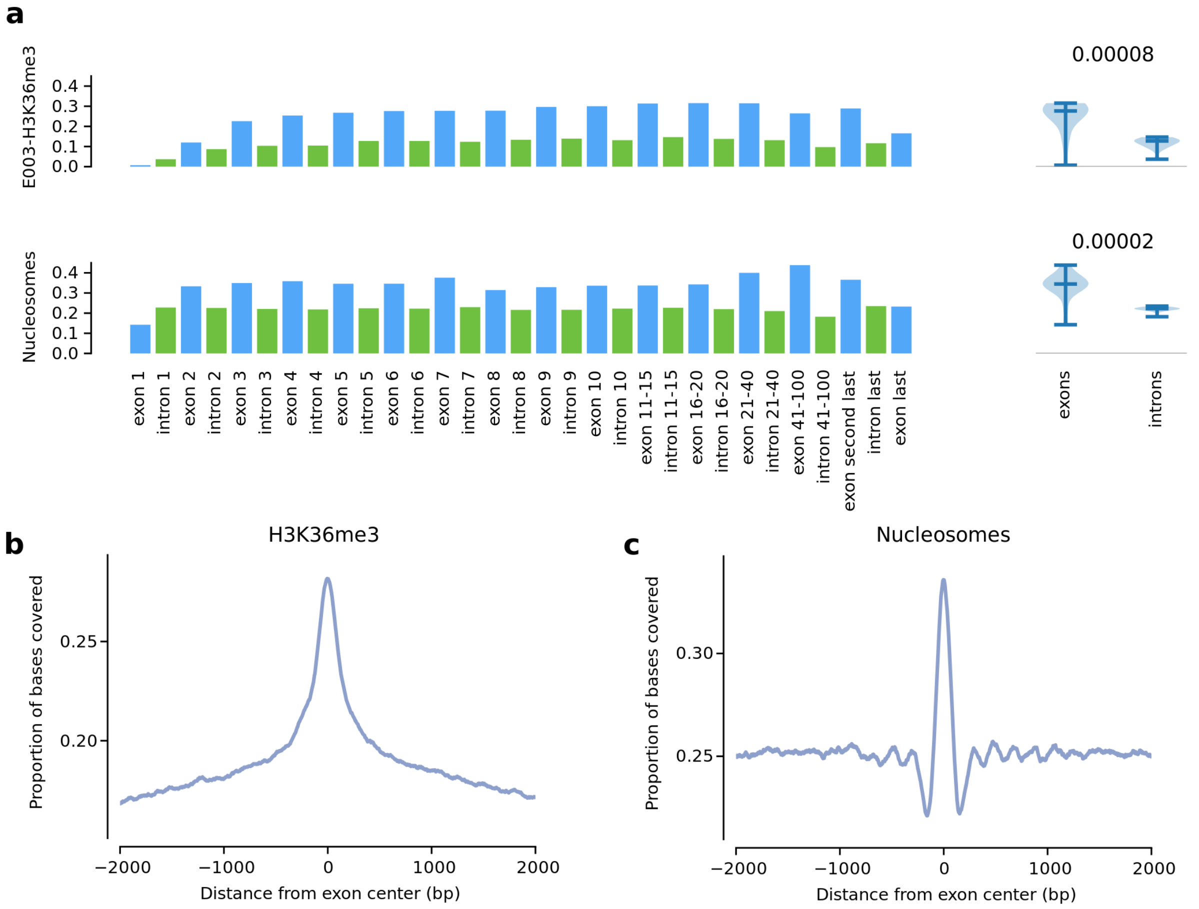

Supplementary Figure 7. Exonic enrichment for the H3K36me3 mark and nucleosomes. Exonic and intronic coverage of H3K36me3 peaks in the H1-hESC embryonic stem cell line (E003) and of nucleosome-covered regions in GM12878 (lymphoblastoid cell line). **(a)** At the left, each bar represents the coverage of the mark in exons or introns at different positions of gene structure. At the right, violin plots show the distribution of the exonic and intronic coverage of each chromatin feature across the gene structure. The p-value from a two-tailed Mann–Whitney test compares the two distributions. Note that most of the difference comes from internal exons compared to flanking intronic sequences. **(b)** Proportion of bases covered by H3K36me3 across internal exons and flanking introns along a middle exon-centered 4001-nt window. **(c)** Proportion of bases covered by nucleosomes across internal exons and flanking introns along a middle exon-centered 4001-nt window.

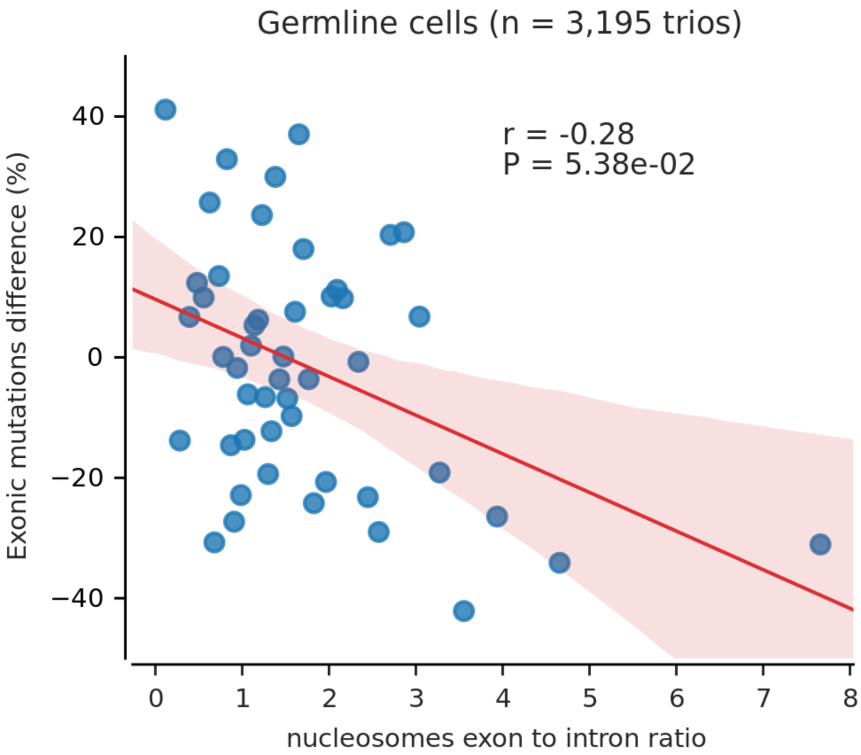

Supplementary Figure 8. Deviation in the exonic mutation burden as a function of the nucleosome exon-to-intron ratio. Blue dots denote 50 groups of genes binned by their exon-to-intron ratio of nucleosome density (x-axis), which was derived from the lymphoblastoid cell line GM12878. The relative difference between the total observed and expected number of exonic mutations per group is shown on the y-axis. Only mutations from healthy probands were used and the expectation was computed using a 3-mer model. The trendline and its confidence interval were added using the seaborn package of Python, while the correlation coefficient and its significance were computed using the same iteratively re-weighted least-squares approach as used by Frigola et al.[8] to ensure comparability.

Supplementary Tables

Supplementary Table 1. Proportion of sequences filtered by genome mappability issues

**Genomic regions**	**affected bp [%]**	**affected windows [%]**
UCSC blacklisted regions (low mappability)	0.01	0.04
hg19 low complexity (repetitive) regions	0.68	22.51
Intersection	0.69	22.54

Middle exon-centered 2001-nt sequences with at least one affected nucleotide are classified as affected.

Supplementary Table 2. Technical characteristics of the DNM datasets

Dataset	Sequencing technology	Mean Cov.	Calling pipeline	Quality controls
GoNL[1]	Hiseq2000	∼13x	GATK (PhaseByTransmission)	a,d,e,f
Sasani[2]	HiseqX	∼30x	GATK	b
Goldmann2016[3]	Complete Genomics	∼60x	cga-tools	a,d
Goldmann2018[4]	Hiseq2000	∼40x	GATK (joint HaplotypeCaller, PhaseByTransmission and ReadBackPhasing)	a,c,d
Yuen[5*]	Hiseq2000 (n=561)	∼34x	DenovoGear + GATK	d
	Complete Genomics (n=1,233)	∼54.5x	cga-tools + custom method	d
	HiseqX (n=3,411)	∼36.4x	DenovoGear + GATK	d
Halldorsson[6]	GA II, Hiseq and HiseqX	∼30x	Graphtyper	b,c
An[7]	Hiseq2000 (n=40), HiseqX (n=1,862)	∼35.5x	TrioDeNovo + DenovoGear + PlinkSeq + DenovoFlow	e

[*] Authors found no differences in DNM calling across sequencing platforms.

(a) Random Forest classifier based on sequencing validated DNM candidates.

(b) Validation through the transmission of DNM candidates across 3 generation pedigrees.

(c) Validation through monozygotic-twin pairs.

(d) Validation through Sanger sequencing.

(e) Validation through Illumina Miseq sequencing.

(f) Validation through Ion Torrent sequencing.

Supplementary Table 3. Excess in exonic burden across mutations from different conditions stratified by mutation class

**Condition**	**Mutation**	**Obs. exonic**	**Exp. exonic**	**Exonic excess [%]**	**Emp. p-value**
Healthy	Synonymous	404	409	-1.2 ± 4.7	0.391
	Nonsynonymous	1085	1060	2.4 ± 2.8	0.194
Autism	Synonymous	406	408	-0.6 ± 4.7	0.476
	Nonsynonymous	1241	1111	11.7 ± 3.2	0.001

Errors of the exonic excess denote one s.d. from 1000 permutations (**Methods**).

Supplementary Table 4. Extended sequence context dependency for Goldmann et al. (2018)[4] stratified by mutation class

Model	Class	Exonic excess [%]	Emp. p-value	Log-likelihood	Param.	AIC
1-mer	Synonymous	10.3 ± 9.5	0.130	−65704	12	131432
	Nonsynonymous	19.6 ± 6.3	0.001	−67336	12	134697
CpG	Synonymous	-3.2 ± 7.9	0.679	−64306	18	128648
	Nonsynonymous	3.4 ± 7.9	0.260	−65871	18	131777
3-mer	Synonymous	-2.8 ± 7.9	0.644	−64037	192	128457
	Nonsynonymous	4.3 ± 4.7	0.201	−65592	192	131569
5-mer	Synonymous	-2.0 ± 7.9	0.595	−63796	1344	130279
	Nonsynonymous	4.4 ± 6.3	0.193	−65333	1344	133353
7-mer	Synonymous	0.3 ± 7.9	0.500	−63726	2496	132443
	Nonsynonymous	3.5 ± 6.3	0.248	−65257	2496	135506

Errors of the exonic excess denote one s.d. from 1000 permutations (**Methods**).

Supplementary Table 5. Extended sequence context dependency for Halldorsson et al.[6] stratified by mutation class

Model	Class	Exonic excess [%]	Emp. p-value	Log-likelihood	Param.	AIC
1-mer	Synonymous	11.4 ± 6.7	0.027	−139495	12	279014
	Nonsynonymous	36.0 ± 4.9	0.001	−143562	12	287148
CpG	Synonymous	-3.4 ± 5.3	0.744	−135475	18	270986
	Nonsynonymous	16.7 ± 3.8	0.001	−139299	18	278633
3-mer	Synonymous	-2.1 ± 5.6	0.656	−134846	192	270076
	Nonsynonymous	17.1 ± 3.9	0.001	−138645	192	277674
5-mer	Synonymous	-0.9 ± 5.4	0.574	−134329	1344	271347
	Nonsynonymous	17.2 ± 3.9	0.001	−138100	1344	278887
7-mer	Synonymous	0.2 ± 5.9	0.499	−134214	2496	273420
	Nonsynonymous	16.7 ± 3.9	0.001	−137983	2496	280957

Errors of the exonic excess denote one s.d. from 1000 permutations (**Methods**).

Supplementary Table 6. Extended sequence context dependency for data pooled across all cohorts stratified by mutation class

Model	Class	Exonic excess [%]	Emp. p-value	Log-likelihood	Param.	AIC
1-mer	Synonymous	13.0 ± 3.2	0.001	−483500	12	967023
	Nonsynonymous	28.3 ± 3.2	0.001	−495583	12	991189
CpG	Synonymous	−2.1 ± 3.2	0.786	−468484	18	937005
	Nonsynonymous	9.8 ± 1.6	0.001	−479643	18	959323
3-mer	Synonymous	−1.1 ± 3.2	0.651	−466394	192	933172
	Nonsynonymous	10.4 ± 1.6	0.001	−477517	192	955418
5-mer	Synonymous	−0.7 ± 3.2	0.592	−464468	3072	935081
	Nonsynonymous	9.7 ± 1.6	0.001	−475513	3072	957169
7-mer	Synonymous	−0.5 ± 3.2	0.561	−462879	21504	968766
	Nonsynonymous	8.6 ± 1.6	0.001	−473870	21504	990748

Errors of the exonic excess denote one s.d. from 1000 permutations (**Methods**).

Supplementary References

1. Francioli, L. C. *et al.* Genome-wide patterns and properties of de novo mutations in humans. *Nature Genetics* **47,** 822–826. ISSN: 15461718 (2015).
2. Sasani, T. A. *et al.* Large, three-generation human families reveal post-zygotic mosaicism and variability in germline mutation accumulation. *eLife* **8,** 1–24. ISSN: 2050084X (2019).
3. Goldmann, J. M. *et al.* Parent-of-origin-specific signatures of de novo mutations. *Nature Genetics* **48,** 935–939. ISSN: 15461718 (2016).
4. Goldmann, J. M. *et al.* Germline de novo mutation clusters arise during oocyte aging in genomic regions with high double-strand-break incidence. *Nature Genetics* **50,** 487–492. ISSN: 15461718 (2018).
5. Yuen, R. K. *et al.* Whole genome sequencing resource identifies 18 new candidate genes for autism spectrum disorder. *Nature Neuroscience* **20,** 602–611. ISSN: 15461726 (2017).
6. Halldorsson, B. V. *et al.* Characterizing mutagenic effects of recombination through a sequence-level genetic map. *Science* **363.** ISSN: 10959203. doi:`10.1126/science.aau1043` (2019).
7. An, J.-Y. *et al.* Genome-wide de novo risk score implicates promoter variation in autism spectrum disorder. *Science* **362.** ISSN: 0036-8075. doi:`10.1126/science.aat6576` (2018).
8. Frigola, J. *et al.* Reduced mutation rate in exons due to differential mismatch repair. *Nature Genetics* **49,** 1684–1692. ISSN: 15461718 (2017).
