# Supplementary material for: Germline de novo mutation rates on exons versus introns in humans
Source: Nat Commun. 2020 Jul 3;11:3304. doi: 10.1038/s41467-020-17162-z (PMC7334200; doi:10.1038/s41467-020-17162-z)
Supplement: Supplementary file 3 — Reporting Summary [file 41467_2020_17162_MOESM3_ESM.pdf]

nature research

Corresponding author(s): Donate Weghorn & Antonio Barbadilla

Last updated by author(s): 05/21/2020

Reporting Summary

Nature Research wishes to improve the reproducibility of the work that we publish. This form provides structure for consistency and transparency in reporting. For further information on Nature Research policies, see Authors & Referees and the Editorial Policy Checklist .

Statistics

For all statistical analyses, confirm that the following items are present in the figure legend, table legend, main text, or Methods section.

n/a	Confirmed	
☐	☒	The exact sample size (*n*) for each experimental group/condition, given as a discrete number and unit of measurement
☐	☒	A statement on whether measurements were taken from distinct samples or whether the same sample was measured repeatedly
☐	☒	The statistical test(s) used AND whether they are one- or two-sided<br>*Only common tests should be described solely by name; describe more complex techniques in the Methods section.*
☐	☒	A description of all covariates tested
☐	☒	A description of any assumptions or corrections, such as tests of normality and adjustment for multiple comparisons
☐	☒	A full description of the statistical parameters including central tendency (e.g. means) or other basic estimates (e.g. regression coefficient) AND variation (e.g. standard deviation) or associated estimates of uncertainty (e.g. confidence intervals)
☐	☒	For null hypothesis testing, the test statistic (e.g. *F*, *t*, *r*) with confidence intervals, effect sizes, degrees of freedom and *P* value noted<br>*Give P values as exact values whenever suitable.*
☒	☐	For Bayesian analysis, information on the choice of priors and Markov chain Monte Carlo settings
☒	☐	For hierarchical and complex designs, identification of the appropriate level for tests and full reporting of outcomes
☐	☒	Estimates of effect sizes (e.g. Cohen's *d*, Pearson's *r*), indicating how they were calculated

Our web collection on statistics for biologists contains articles on many of the points above.

Software and code

Policy information about availability of computer code

Data collection	Published datasets were collected from the official repositories and in one case by direct request to the authors. They were subsequently processed according to the descriptions in the Methods section. The predicted consequence class of all DNMs was obtained using the Ensembl Variant Effect Predictor (VEP) for the GRCh37/hg19 assembly.
Data analysis	We used code that was published in Frigola et al. (2017), in pure or slightly adapted form. We also used custom scripts for data analysis according to the descriptions in the Methods section. All used code is deposited at https://bitbucket.org/weghornlab/germline_intron_exon_mutrate/src/master/.

For manuscripts utilizing custom algorithms or software that are central to the research but not yet described in published literature, software must be made available to editors/reviewers. We strongly encourage code deposition in a community repository (e.g. GitHub). See the Nature Research guidelines for submitting code & software for further information.

Data

Policy information about availability of data

All manuscripts must include a data availability statement. This statement should provide the following information, where applicable:

- Accession codes, unique identifiers, or web links for publicly available datasets
- A list of figures that have associated raw data
- A description of any restrictions on data availability

Mutation data from the Genomes of the Netherlands (GoNL) project was downloaded from [http://www.nlgenome.nl]. The remaining mutation datasets were either by direct request to the authors (Goldmann et al., 2018) or downloaded from the supplementary tables of their respective publications (Goldmann et al., 2016; Halldorsson et al., 2019; An et al., 2018; Yuen et al., 2017; Sasani et al., 2019). Coordinates of unreliable regions were obtained from the UCSC Genome Browser, available at [http://genome.ucsc.edu/cgi- bin/hgFileUi?db=hg19&g=wgEncodeMapability]. Narrow peak coordinates and genome-wide read- coverage of H3K36me3 from human embryonic stem cell H1-hESC (E003) were downloaded through the Epigenome Roadmap consortium data portal [http://www.roadmapepigenomics.org/data]. The genome-wide nucleosome positioning density graph of ENCODE42 cell line GM12878 (lymphoblastoid cell line) was obtained via the UCSC genome browser [https://hgdownload.soe.ucsc.edu/downloads.html].

Field-specific reporting

Please select the one below that is the best fit for your research. If you are not sure, read the appropriate sections before making your selection.

☒ Life sciences ☐ Behavioural & social sciences ☐ Ecological, evolutionary & environmental sciences

For a reference copy of the document with all sections, see nature.com/documents/nr-reporting-summary-flat.pdf

Life sciences study design

All studies must disclose on these points even when the disclosure is negative.

Sample size	Sample size was given by the size of the available published de novo mutation datasets.
Data exclusions	Genomic regions with low mappability were filtered out in the gene-by-gene analysis and to test for robustness in Supplementary Figure 2, as detailed in the Methods section.
Replication	All analyses can be repeated by others. Since no experiments were carried out, the concept of replication does not apply.
Randomization	There was no case-control scenario or allocation of samples into groups.
Blinding	n/a

Reporting for specific materials, systems and methods

We require information from authors about some types of materials, experimental systems and methods used in many studies. Here, indicate whether each material, system or method listed is relevant to your study. If you are not sure if a list item applies to your research, read the appropriate section before selecting a response.

Materials & experimental systems

n/a	Involved in the study	
☒	☐	Antibodies
☒	☐	Eukaryotic cell lines
☒	☐	Palaeontology
☒	☐	Animals and other organisms
☒	☐	Human research participants
☒	☐	Clinical data

Methods

n/a	Involved in the study	
☒	☐	ChIP-seq
☒	☐	Flow cytometry
☒	☐	MRI-based neuroimaging
