# Supplementary material for: Germline de novo mutation rates on exons versus introns in humans
Source: Nat Commun. 2020 Jul 3;11:3304. doi: 10.1038/s41467-020-17162-z (PMC7334200; doi:10.1038/s41467-020-17162-z)
Supplement: Supplementary file 2 — Peer Review [file 41467_2020_17162_MOESM2_ESM.pdf]

Reviewers' comments:

Reviewer #1 (Remarks to the Author):

This paper describes an analysis of published de novo mutation datasets to test the hypothesis that the exonic mutation rate is different from the intronic mutation rate in the germline. This hypothesis was first posited by Frigola et al in a paper in 2017, following observation of this effect in the somatic cells of a cohort of colorectal tumours. It is an important hypothesis to test because, if true, it would undermine many evolutionary genetics models that assume that mutation rate is independent of coding status of the DNA sequence, so that any difference in diversity can be interpreted as differences in the action of population genetic processes following mutation, such as selection.

The authors here assembled a large amount of data and tested this hypothesis rigorously. I have very few comments on the science – they have approached the topic logically and have explored it thoroughly. The paper is clearly written. The result is clearly in line with previous expectations and the hypothesis proposed by Frigola is not only not supported, but is effectively refuted by the data and the analyses. This is a very important result for molecular evolutionary and population genetics.

I have some minor comments.
1. Page 2 para 3. POLE should be defined and explained at the first mention.
2. Page 2 para 3. "mutation rates are surprisingly lower". Surprisingly should be removed or be placed at the start of the sentence (replacing "Recently") – at the moment the placing of "surprisingly" suggests that the magnitude of the drop in exonic mutation rate is surprising, "surprisingly lower", rather than the actual fact of the mutation rate being lower at all being surprising.
3. Page 3 para 1. "Differential" should be "different".
4. Page 3 para 3. After "In contrast" it should read "In contrast, in our study, the observed germline..." to prevent ambiguity and emphasise that you are referring to this study not the study by Frigola et al discussed in the previous sentence.
5. Page 11 para 1. Coordinates of exonic and intronic boundaries after mappability filtering should either be available as a supplementary txt file hosted by the journal, or hosted by figshare or similar stable open access repository.
5. Page 11 Code availability. These scripts should be made openly accessible on Github or Figshare or a suitable open access repository.

Reviewer #2 (Remarks to the Author):

Rodriguez-Galindo et al. investigated whether there is an elevated mutation rate in exons compared with that in introns. This study was conducted after Frigola et al 2017 showed that there is a reduced mutation rate in exons compared with introns in somatic mutations in tumors, which the authors showed that it would be due to enhanced MMR activities in exons. In this study, the authors showed that there is no evidence of higher mutation rates in exons, and that in mixed disease cohorts, there is an increased mutation rate in nonsynonymous mutation rates compared with the intron mutation rate.

This study is particularly crucial because evolutionary models are based on the assumption that the base mutation rate is the same between the two, except for those variants that are subject to positive or negative selection. The authors gave strong evidence that the assumption holds in germline mutations. The article is well written and easy to follow. While I have some concerns about the differences in the results in the different cohorts, I would recommend this manuscript to be published with some additional analysis.

Firstly, as the authors showed in Table 1, the point estimates for exon excess compared with intron are mostly positive, although most of them are insignificant. They showed that when they stratify the

exonic mutations by synonymous and nonsynonymous, only nonsynonymous mutations showed excess mutations compared with expectation. They further showed that this is the case in the Autism probands but not the "healthy" probands.
The authors hypothesize that it could be due to ascertainment bias from disease probands being more likely to carry functional impactful variants (Table S3). I think that the disease cohort may exhibit a slight increase in the number of functional variants, but 10.5% excess seems pretty substantial. Can the authors also look into the largest single cohort, Halldorsson dataset, which consists of multiple diseases, does it also exhibit such trend in non-synonymous and synonymous mutations?

Secondly, in Frigola et al. 2017, after filtering and removing cancer driving genes, there are 12,104 genes left (including X, Y); in this manuscript, after filtering (and removing X, Y), there are 12,754 genes left. Assuming the way of constructing the meta-exon is the same (which brings me to the final point that data and scripts need to be shared), could the difference in the observation partially be explained by the differences in the genes selected? 1) Can the authors take the intersect between the two sets of genes and repeat the analysis? 2) show the distribution of exon vs. intron mutation rate to see if the excess mutations in exons is driven by a few genes.

Finally, the authors claimed that custom scripts for data analysis are available upon request from the authors. I think the key scripts and processed data need to be released.

Minor comments:

On page 33, Table S3. the calling of Complete Genomics variants in the Yuen dataset does not involve GATK, but uses a heuristic method described in
Yuen RKC, Thiruvahindrapuram B, Merico D, Walker S, Tammimies K, Hoang N, et al. Whole-genome sequencing of quartet families with autism spectrum disorder. Nat Med. 2015;21: 185–191.

Wendy Wong

Point-by-point response to reviewers

Reviewer #1

1. Page 2 para 3. POLE should be defined and explained at the first mention.
2. Page 2 para 3. "mutation rates are surprisingly lower". Surprisingly should be removed or be placed at the start of the sentence (replacing "Recently") – at the moment the placing of "surprisingly" suggests that the magnitude of the drop in exonic mutation rate is surprising, "surprisingly lower", rather than the actual fact of the mutation rate being lower at all being surprising.
3. Page 3 para 1. "Differential" should be "different".
4. Page 3 para 3. After "In contrast" it should read "In contrast, in our study, the observed germline..." to prevent ambiguity and emphasise that you are referring to this study not the study by Frigola et al discussed in the previous sentence.
5. Page 11 para 1. Coordinates of exonic and intronic boundaries after mappability filtering should either be available as a supplementary txt file hosted by the journal, or hosted by figshare or similar stable open access repository.
6. Page 11 Code availability. These scripts should be made openly accessible on Github or Figshare or a suitable open access repository.

We thank Reviewer #1 for their comments. We have incorporated into the new version of the manuscript corrections 1-4. Regarding corrections 5-6, we have uploaded and documented all the code and intermediate files (including coordinates after filtering) on bitbucket:
https://bitbucket.org/weghornlab/germline_intron_exon_mutrate

Reviewer #2

Firstly, as the authors showed in Table 1, the point estimates for exon excess compared with intron are mostly positive, although most of them are insignificant. They showed that when they stratify the exonic mutations by synonymous and nonsynonymous, only nonsynonymous mutations showed

excess mutations compared with expectation. They further showed that this is the case in the Autism probands but not the ”healthy” probands.

The authors hypothesize that it could be due to ascertainment bias from disease probands being more likely to carry functional impactful variants (Table S3). I think that the disease cohort may exhibit a slight increase in the number of functional variants, but 10.5% excess seems pretty substantial. Can the authors also look into the largest single cohort, Halldorsson dataset, which consists of multiple diseases, does it also exhibit such trend in non-synonymous and synonymous mutations?

We thank Reviewer #2 for their comments. To address this first question, we repeated the analysis exclusively for the Halldorsson et al. (2019) dataset across different context dependency models, and separately for synonymous and non-synonymous variants (as had been done in the manuscript for the Goldmann et al., 2018, dataset and the joint dataset in Supplementary Tables S4 and S5, respectively). Except for the 1-mer model, which we know highly underestimates mutations in exons, all models show a significant excess in nonsynonymous mutations, in contrast to no significant excess for synonymous variants (**Table 1**). We have added this table as the new Supplementary Table S5 to the manuscript.

We agree with Reviewer #2 that the nonsynonymous excess seems large. That is why we had already contacted the authors of the Halldorsson et al. (2019) study to request extra information about the proportion of diseased individuals and, ideally, the complete list of phenotypes for each proband of the dataset in order to separate by healthy and diseased and redo the analysis as an extra sanity check. Unfortunately, the authors expressed the impossibility to share this information due to data protection conflicts. In summary, ruling out the mutational process and positive selection in the germline (a preposterous hypothesis) as possible sources of this excess of nonsynonymous variation, the ascertainment bias seems the most plausible explanation for this pattern across datasets.

Table 1. **Extended sequence context dependency for Halldorsson et al. (2019), stratified by mutation class. Errors denote one s.d.**

Model	Class	% increase	Emp. p-value	Log-likelihood	Param.	AIC
1-mer	Synonymous	11.4 ± 6.7	0.027	−139495.1	12	279014.2
	Nonsynonymous	36.0 ± 4.9	0.001	−143561.9	12	287147.8
CpG	Synonymous	-3.4 ± 5.3	0.744	−135474.9	18	270985.9
	Nonsynonymous	16.7 ± 3.8	0.001	−139298.7	18	278633.3
3-mer	Synonymous	-2.1 ± 5.6	0.656	−134846.2	192	270076.3
	Nonsynonymous	17.1 ± 3.9	0.001	−138644.9	192	277673.8
5-mer	Synonymous	-0.9 ± 5.4	0.574	−134329.4	1152	270962.8
	Nonsynonymous	17.2 ± 3.9	0.001	−138099.7	1152	278503.5
7-mer	Synonymous	0.2 ± 5.9	0.499	−134214.0	2304	273036.1
	Nonsynonymous	16.7 ± 3.9	0.001	−137982.6	2304	280573.2

Secondly, in Frigola et al. 2017, after filtering and removing cancer driving genes, there are 12,104 genes left (including X, Y); in this manuscript, after filtering (and removing X, Y), there are 12,754 genes left. Assuming the way of constructing the meta-exon is the same (which brings me to the final point that data and scripts need to be shared), could the difference in the observation partially be explained by the differences in the genes selected? 1) Can the authors take the intersect between the two sets of genes and repeat the analysis? 2) show the distribution of exon vs. intron mutation rate to see if the excess mutations in exons is driven by a few genes.

In contrast to the study by Frigola et al. (2017), the set of genes we use includes known cancer driver genes, because a priori we do not expect a selection signal on these genes in the germline. Conversely, we excluded genes on sexual chromosomes, where we know that *de novo* mutation calling is less reliable. To address the comments of the Reviewer, we performed the internal exon-centered analysis with the intersection between the two sets of genes, that is, excluding cancer driver genes and overlapping genes plus filtering genes on sexual chromosomes. This leaves a total of 10,216 genes for analysis. We find that the results do not change substantially, with a significant excess of exonic mutations of 6.8% (P=0.001), compared to 7.2% (P=0.001) in the manuscript. The corresponding

plot is shown in **Figure 1**. We leave the decision to the editor whether to include this additional comparison in the manuscript.

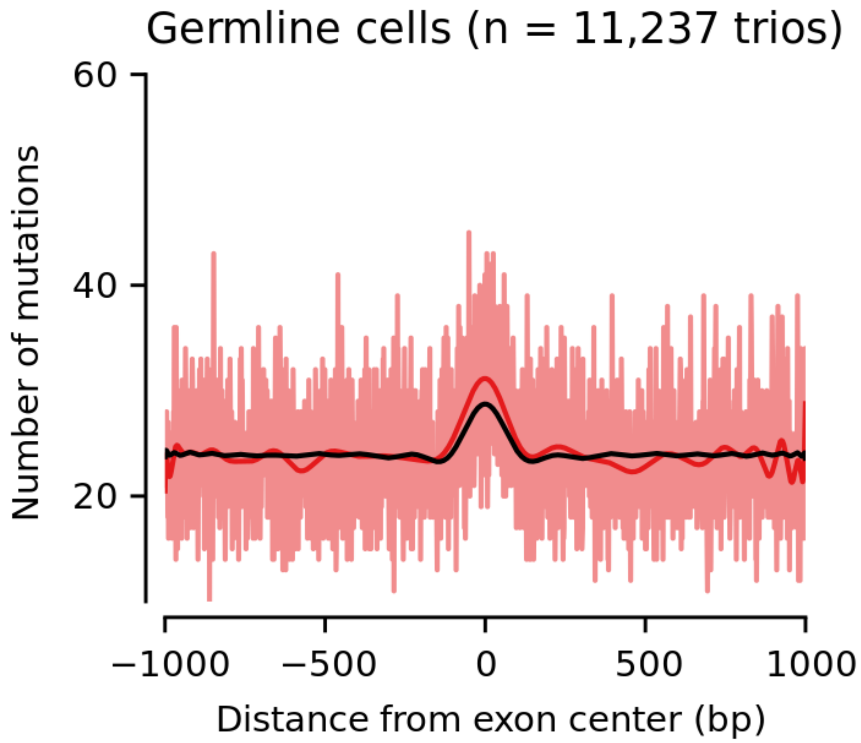

Figure 1. Internal exon-centered analysis for germline *de novo* mutations from the intersection set of genes. Exon-centered 2,001-nt-wide observed and expected mutational profiles. The light red line represents the observed number of mutations at each position, whereas the dark red and black lines represent smoothed numbers of observed and expected mutations, respectively, obtained from a polynomial fit. Only windows from genes which were contained in both the set used by Frigola et al. (2017) and our set of genes were used. The observed germline exonic mutation burden is significantly increased by 6.8% (P=0.001, permutation-based test) compared to the expectation.

Finally, the authors claimed that custom scripts for data analysis are available upon request from the authors. I think the key scripts and processed data need to be released.

With regard to the way meta-exon are extracted, we used exactly the same script and annotation as Frigola et al. (2017), but skipping the filtering step with regard to cancer driver genes and filtering sexual chromosome coordinates at the end of the pipeline. As suggested by the Reviewer, we have uploaded and documented all the code and intermediate files on bitbucket, https://bitbucket.org/weghornlab/germline_intron_exon_mutrate.

Minor comments:

On page 33, Table S3. the calling of Complete Genomics variants in the Yuen dataset does not involve GATK, but uses a heuristic method described in Yuen RKC, Thiruvahindrapuram B, Merico D, Walker S, Tammimies K, Hoang N, et al. Whole-genome sequencing of quartet families with autism spectrum disorder. Nat Med. 2015;21: 185–191.

We corrected the error pointed out regarding Supplementary Table S3 about the calling of Complete Genomics variants in the Yuen dataset.

References

J. Frigola et al. "Reduced mutation rate in exons due to differential mismatch repair". Nature Genetics 49.12 (2017), pp. 1684-1692.

J. M. Goldmann et al. "Germline de novo mutation clusters arise during oocyte aging in genomic regions with high double-strand-break incidence". Nature Genetics 50.4 (2018), pp. 487-492.

B. V. Halldorsson et al. "Characterizing mutagenic effects of recombination through a sequence-level genetic map". Science 363.6425 (2019).

REVIEWERS' COMMENTS:

Reviewer #2 (Remarks to the Author):

The authors have addressed all my concerns and I have no further comments. Nice work!

Wendy
